# Genome characterization of a begomovirus isolated from Brazilian *Carica papaya* plants

**DOI:** 10.1007/s00705-026-06759-2

**Published:** 2026-10-16

**Authors:** Yam Sousa Santos, Eliza F. M. Bellard do Nascimento, Caroline Bezerra, Viviana Ramírez-Rios, Andreza Henrique Vidal, Jhon Eric Alves Fernandes, Fernando L. Melo, Paolo Margaria, Rosana Blawid, Emanuel Felipe Medeiros Abreu, Simone Graça Ribeiro

**Affiliations:** 1 Embrapa Recursos Genéticos e Biotecnologia, Brasília, 70770-917 DF Brazil; 2 Plant Virus Department, Leibniz Institute DSMZ - German Collection of Microorganisms and Cell Cultures GmbH, Inhoffenstrasse 7B, 38124 Braunschweig, Germany; 3 Departamento de Agronomia, Universidade Federal Rural de Pernambuco, Recife, 52171-900 PE Brazil

**Keywords:** Plant virus, *Geminiviridae*, Emerging viruses

## Abstract

Papaya (*Carica papaya* L.) plants are susceptible to several viral pathogens. However, information regarding papaya-associated DNA viruses in Latin America is limited. In this study, we collected symptomatic papaya plants in Baraúna, Rio Grande do Norte, Brazil, and screened for begomovirus infection. Three of ten samples tested positive through PCR using universal primers, and were further analyzed using rolling circle amplification and Oxford Nanopore sequencing. This approach allowed us to recover complete circular viral genomes with the typical DNA-A segment organization of begomoviruses. We named this newly identified viral agent papaya mottle virus (PaMoV). No DNA-B component was detected in the samples, suggesting that PaMoV is a monopartite begomovirus. The sequences from the three isolates shared over 95% nucleotide identity. Pairwise sequence identity along with phylogenetic analyses supported the classification of PaMoV as a member of a novel begomovirus species, for which we propose the name “*Begomovirus papayavariati*”. This is the first report of a begomovirus associated with *C. papaya* in Brazil.

Papaya (*Carica papaya* L.) is one of the most widely cultivated tropical fruit crops, valued for its nutritional content and economic importance across tropical and subtropical regions [1]. Despite its global relevance, papaya production is affected by viral diseases, which represent a major cultivation challenge [2]. Among these viral agents, papaya ringspot virus (PRSV; *Potyvirus papayanuli*, family *Potyviridae*) and the papaya meleira virus complex associated with two fusagraviruses (*Fusagravirus roku* and *Fusagravirus kyu*, family *Fusagraviridae*) and different umbra-like viruses, are considered the most economically important viruses due to their substantial effects on plant development and fruit quality [3–5]. Papaya is also susceptible to DNA viruses belonging to the family *Geminiviridae*, which are distinguished by single-stranded DNA genomes and the ability to infect a wide range of cultivated and wild plant species worldwide. Numerous viruses belonging to the *Begomovirus* genus are associated with papaya leaf curl disease, a condition marked by symptoms such as leaf curling, vein thickening, stunted growth, and decreased fruit yield [6–8]. These viruses impact the papaya crop primarily in India and other Asian countries. In the American continent, there are few reports of begomoviruses associated with papaya, mainly in Mexico and the USA [9–11]. These findings suggest that those viruses are an underexplored component of the papaya virome in the Americas. Despite Brazil being one of the world’s major papaya-producing countries, no begomoviruses have been reported infecting papaya in the country, indicating a potential gap in viral surveillance for this crop. To address this gap, we conducted a survey of papaya plants in Northeastern Brazil to assess their possible infection with begomoviruses. Therefore, in this study, we applied a rapid PCR-based screening strategy using universal degenerate primers, followed by Oxford Nanopore sequencing of rolling circle amplification (RCA) products. This approach allowed us to recover the complete genome sequence of a previously undescribed begomovirus associated with symptomatic *C. papaya* plants.

Ten plants with symptoms typical of viral disease, such as leaf mottling, chlorotic spots, and leaf deformation, were collected from a commercial field in Baraúna, Rio Grande do Norte, Brazil (Fig. 1), and total DNA was extracted using a CTAB-based protocol [12]. Samples were screened for begomoviruses by PCR using the universal degenerate primers PAL1v1978/PAR1c496 to amplify a 1.1 kb fragment spanning the common region and adjacent portions of the AC1 and AV1 ORFs of the DNA-A component [13]. Three of the ten samples (N20, N21, and N23) tested positive and were then subjected to RCA as described previously [14]. Additional PCR assays using universal degenerated primers [13] for begomovirus DNA-B segment were performed, however no amplicons were obtained. Individual RCA products were sequenced using Oxford Nanopore Technology (ONT, Oxford, UK). Barcoded libraries were prepared with the Rapid Sequencing DNA V14 Barcoding Kit (SQK-RBK114.96), loaded onto an FLO-PRO114M flow cell, and sequenced on the PromethION P2 Solo platform. Raw sequencing data were basecalled with Dorado v2.0.0 using the dna_r10.4.1_e8.2_400bps_sup@v5.2.0 model.

**Fig. 1:**
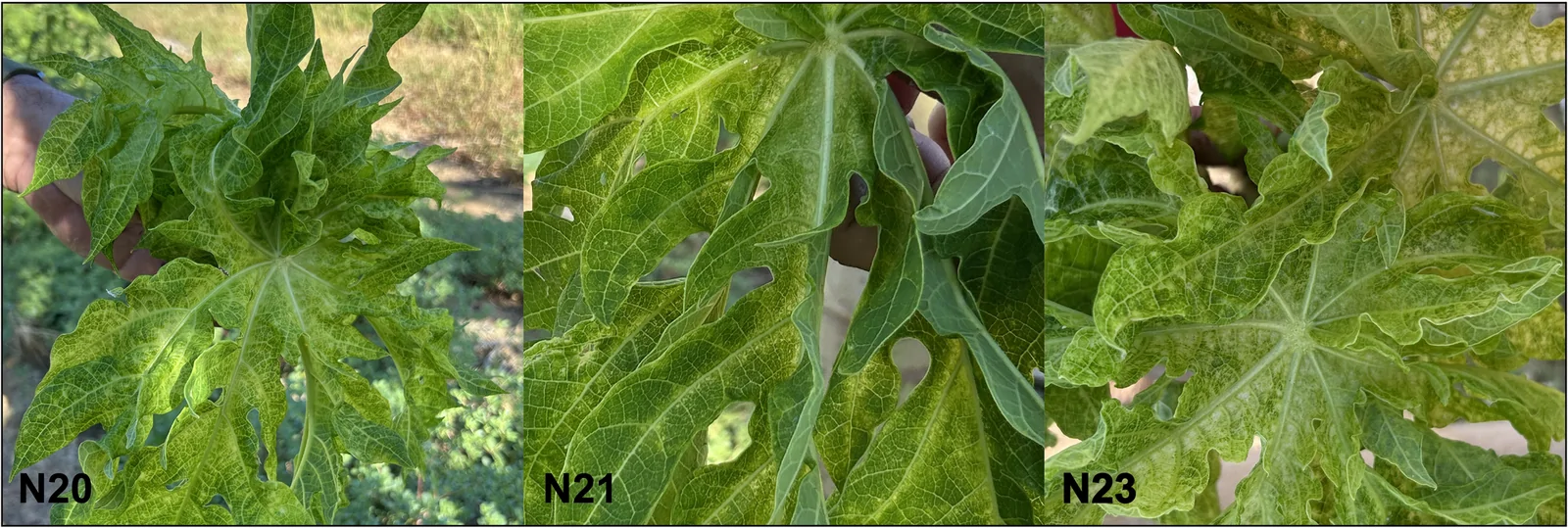
Symptomatic papaya leaves collected in Baraúna, Rio Grande do Norte, Brazil. *Carica papaya* leaves exhibiting leaf mottling, chlorotic spots, and leaf deformation in field-collected samples. Each sample is identified by its corresponding code

The sequencing runs for samples N20, N21, and N23 produced 12,185, 39,918, and 22,091 reads, respectively. Putative circular viral genome sequences were identified by repeated concatemeric units within individual raw reads, a pattern consistent with RCA-mediated amplification of circular DNA molecules. Circular sequences were compared by BLASTn against CRESS DNA virus sequences available in GenBank, and begomovirus-like sequences were retained. Genome organization and open reading frames (ORFs) were predicted using Geneious Prime^®^ 2025.0.3. Retrieved sequences were mapped with Minimap2 [15] to closely related contigs to correct potential sequencing errors based on the consensus sequence at each genomic position. From this analysis, one complete viral genome was recovered per sample, each one of 2637 nt in length. We propose the name “papaya mottle virus” (PaMoV) for the newly discovered viral agent. BLASTn analysis showed that all PaMoV isolates shared the highest nucleotide identity (in the range 84.19% − 84.70%) with isolates of the monopartite tomato leaf curl purple vein virus (ToLCPVV; *Begomovirus solanumviolavenae*; GenBank accessions NC_035481.1, KY196221.1, and KY196217.1), which was previously reported from tomato plants from Guadalupe, Piauí State, Northeast Brazil. On the other hand, identities below 74% and 69% were observed in comparisons with papaya-infecting begomoviruses from Mexico and India, respectively. PaMoV has a genome organization similar to that of ToLCPVV and other monopartite begomoviruses from the Americas in which the AV2 ORF encoding the pre-coat protein is missing (Fig. 2A), in contrast to Old World monopartite viruses that encode a V2 protein involved in movement/silencing suppression activity [6–8]. Moreover, no cognate DNA-B component was detected by either DNA-B-targeted PCR or ONT sequencing, suggesting that PaMoV is likely monopartite. Although New World begomoviruses are predominantly bipartite, an increasing number of monopartite species have been reported in the Americas [16–20], challenging the traditional view that monopartite begomoviruses are associated with the Old World. To taxonomically characterize the PaMoV sequences, the best BLASTn hits were retrieved from GenBank, and pairwise nucleotide identities were calculated using the MUSCLE alignment algorithm implemented in the Sequence Demarcation Tool Version 1.3 (SDT) [21]. The three PaMoV genomes shared identities of 96% and 99% among themselves, consistent with their classification as isolates of the same virus (Fig. 2B). On the other hand, PaMoV genome sequences exhibited less than 85% nt identity to other begomoviruses, supporting their designation as members of a novel species. A phylogenetic relationship was inferred using maximum likelihood in IQ-TREE, with ModelFinder and branch support assessed using ultrafast bootstrap with 1,000 replicates [22]. This analysis showed that PaMoV isolates clustered together, forming a distinct and well supported lineage relative to the closely related ToLCPVV isolates (Fig. 2B).

**Fig. 2:**
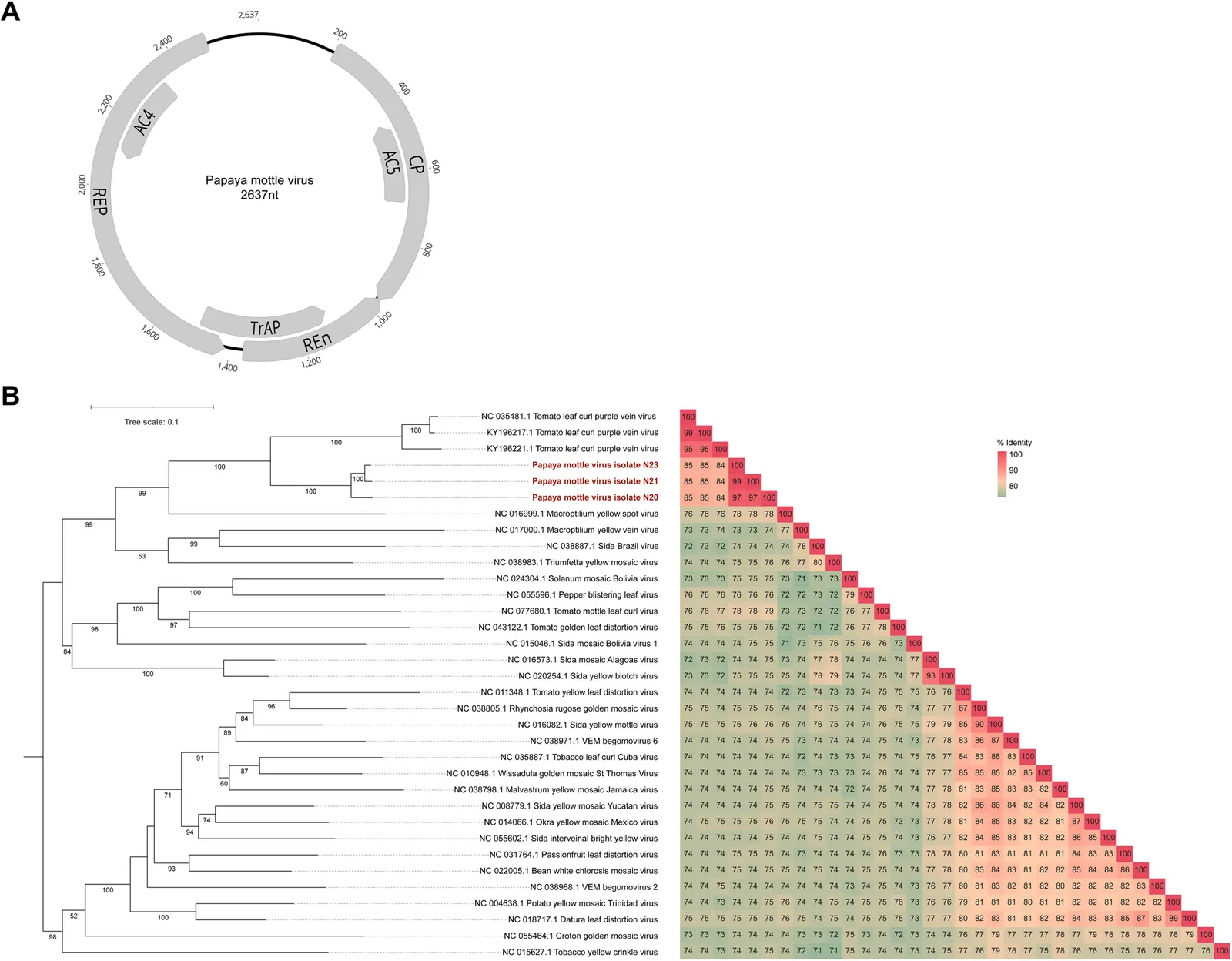
(**A**) Circular genome map showing the open reading frames of a representative PaMoV isolate. Image generated with Geneious Prime^®^ 2025.0.3 software (**B**) Midpoint-rooted maximum likelihood phylogenetic tree, including all PaMoV isolates and representative closely related begomoviruses. Bootstraps values below 50 were omitted. The tree was visualized using the iTOL web server. The pairwise nucleotide identity matrix was generated with SDT using the same sequence dataset

According to current species demarcation criteria for the genus *Begomovirus*, isolates sharing less than 91% nucleotide identity across the complete genome sequence are considered to represent distinct species [23]. In this context, our results support the classification of the PaMoV isolates as members of a novel begomovirus species, for which we propose the name “*Begomovirus papayavariati*”. This is the first report of a begomovirus associated with *C. papaya* plants in Brazil. The absence of a detectable DNA-B component by both PCR and ONT sequencing, together with the close relationship of PaMoV to the monopartite ToLCPVV [17] suggests that PaMoV most likely represents a monopartite New World begomovirus. However, additional studies are necessary to confirm whether the single PaMoV identified component is sufficient to establish a systemic infection. This finding expands the known diversity of papaya-associated begomoviruses and reinforces the emerging view that monopartite begomoviruses may be more widespread in the Americas than previously recognized. Nevertheless, as the plants infected by PaMoV were also coinfected with the papaya meleira virus complex, further research is required to fulfill Koch’s postulates and clarify the association of PaMoV with symptom expression in the leaves.

## Data Availability

The sequences generated in the current study were deposited at GenBank under accession numbers PZ495449, PZ495450, and PZ495451.
